# Editorial: Epstein-Barr virus and its contribution to pediatric tumors

**DOI:** 10.3389/fonc.2022.1026366

**Published:** 2022-09-08

**Authors:** Paola Chabay, Mario Henrique M. Barros

**Affiliations:** ^1^ Laboratory of Molecular Biology, Pathology Division, Multidisciplinary Institute for Investigation in Pediatric Pathologies (IMIPP-CONICET-GCBA), Ricardo Gutiérrez Children’s Hospital, Buenos Aires, Argentina; ^2^ Institute for Pathology, Klinikum Chemnitz, Chemnitz, Germany

**Keywords:** EBV - Epstein-Barr virus, pediatric, lymphoma, Burkitt, coinfection

Since its discovery in 1964 by Anthony Epstein and his co-workers Yvonne Barr and Bert Achong (1), Epstein Barr virus has drawn the attention of tumour virologists, due to its ability to infect and immortalize B-cells (2, 3) as well as to infect a proportion of T-cells during the primary infection, in a way that is not yet well understood (Barros et al.). In most people, the virus infects its host and establishes a latent infection for life as a harmless passenger, in a delicate and fascinating balance with a healthy immune system (4, 5). However in a percentage of individuals, EBV is associated with development of some B-cell neoplasms (as classical Hodgkin lymphoma [cHL] and Burkitt lymphoma [BL]), T-cell neoplasms (as peripheral T-cell lymphoma and extranodal NK/T-cell lymphoma) and epithelial neoplasms (as lymphoepithelial carcinoma) (6, 7). There is a complex interplay between age of acquisition, symptomatic versus asymptomatic primary infection, and the subsequent risk of EBV-associated cancers. When primary infection occurs in adolescents and young adults, they are more likely to experience Infectious Mononucleosis (IM) (3), which is associated with increased risk of developing EBV-associated cHL (8). In children the primary EBV-infection is almost always asymptomatic (3), but not necessarily risk-free. In underdeveloped and developing regions, a high proportion of cHL and BL in children is EBV-associated and probably related to primary EBV-infection (3, 9, 10). In this age group, the mechanisms involved in the oncogenesis are not entirely elucidated and studies focusing specifically on children are still missing.

In this Research Topic, Dr. Münz published a review that discussed how co-infections such as *Plasmodium falciparum*, KSHV and HIV interact with EBV, modify its immune control, and shape its tumorigenesis. The underlying mechanisms reveal new aspects of EBV-associated pathologies and point toward treatment possibilities for their prevention by the human immune system (Münz). The interaction between EBV and maternal HIV infection in Kenyan infants is analysed by the publication of Samayoa-Reyes et al. who suggest that HIV-infected mothers shed more EBV in saliva than HIV-negative mothers. Moreover infants born to HIV-positive mothers are at risk for loss of control of primary EBV infection, as evidenced by higher EBV viral load following primary infection.

In addition, the Research Topic discusses the pathogenesis of one of the most important EBV-associated paediatric tumours, BL. On the one hand, Xian et al. proposed that measurements of EBV-copy number in plasma may be useful in identifying children with endemic BL versus control children. They suggested that the differentiation of high EBV-copy number associated with tumour versus high EBV-copy number associated with asymptomatic *Plasmodium falciparum* infection could be a useful tool in future research. Therefore the authors explored the link between primary infection, viral reactivation and the development of EBV-related malignancies (Xian et al.). On the other hand, Liao et al. explored the molecular epidemiology of EBV variants associated with specific paediatric BL, since genomic variation in EBV is suspected to play a role in the geographical patterns of these EBV-associated cancers. The authors compared phylogenetic patterns of EBV genomes obtained from BL samples in Africa and from tumour and non-tumour samples from elsewhere. They concluded that EBV obtained from BL in Africa is genetically separate from EBV in Asia. Through comprehensive analysis of nucleotide variations in EBV’s LMP-1 gene, they described 12 LMP-1 patterns, two of which (B and G) were found mostly in Asia. Four LMP-1 patterns (A, AB, D, and F) accounted for 92% of EBVs sequenced from BL in Africa. The results identified extensive diversity of EBV and revealed a limited number of variants in BL from Africa, different from those identified in Asia (Liao et al.).

This Research Topic brings together articles that explore the pathogenesis of both EBV infection in children, as well as EBV-associated pediatric tumours. Furthermore, this issue discusses the link between EBV and co-infections, in addition to the molecular epidemiology of EBV variants associated with specific paediatric neoplasia.

We want to dedicate this editorial to Dr Rocio Hassan, who sadly passed away far too early. She was involved in more than 50 publications focused on pediatric oncohematology in Brazil, and stablished valuable collaborations with partners across Latin America to improve scientific skills of researchers and students from the region. Dr Hassan made a great contribution to the understanding of the etiopathogenesis of EBV-associated paediatric lymphomas in Brazil. Her brilliant intellect, dedication, commitment to transfer knowledge and passion for science are profoundly missed by her friends, collaborators and Latin American science.

## Author contributions

PC and MB conceived of the study and its design, wrote the manuscript. All authors read and approved the final manuscript.

## Acknowledgments

The authors want to thank all the authors on the EBV research that submitted manuscripts for this Research Topic.

## Conflict of interest

The authors declare that the research was conducted in the absence of any commercial or financial relationships that could be construed as a potential conflict of interest.

## Publisher’s note

All claims expressed in this article are solely those of the authors and do not necessarily represent those of their affiliated organizations, or those of the publisher, the editors and the reviewers. Any product that may be evaluated in this article, or claim that may be made by its manufacturer, is not guaranteed or endorsed by the publisher.
